# Comparison of butyl-2-cyanocrylate, gelatin-resorcin-formaldehyde (GRF) compound and suture in stabilization of cartilage grafts in rabbits

**DOI:** 10.1016/S1808-8694(15)30036-7

**Published:** 2015-10-19

**Authors:** Heloisa Juliana Zabeu Rossi Costa, Celina Siqueira Barbosa Pereira, Márcio Paulino Costa, Fabrício Sanchez Soga Sanches Fabri, Carmen Lúcia Penteado Lancellotti, José Eduardo Lutaif Dolci

**Affiliations:** a ENT specialist by the Brazilian Society of Otolaryngology; MSc. In Otolaryngology - FCM Santa Casa de São Paulo; PhD student – ENT Department - FCM Santa Casa de São Paulo; Assistant Professor - University Hospital - Medical School - USP.; b PhD in Otolaryngology – Medical School - Santa Casa de São Paulo, Assistant Professor – Department of Morphology – Medical School - Santa Casa de São Paulo.; c PhD in Medicine (Plastic Surgery) - Medical School - USP, Assistant Professor – Department of Plastic Surgery – Medical School - USP.; d 5^th^ year Medical student – Medical School - Santa Casa de São Paulo.; e PhD in Pathology – Medical School - Santa Casa de São Paulo, Assistant Professor – Department of Pathology – Medical School - Santa Casa de São Paulo.; f PhD in Otolaryngology – Medical School - Universidade de São Paulo, Assistant Professor – Department of Otolaryngology – Medical School - Santa Casa de São Paulo. Santa Casa de Misericórdia de São Paulo.

**Keywords:** butyl-2-cyanoacrylate, compound gelatin-resorcin-formaldehyde, GRF glue, grafts, cartilage, rabbits, tissue glue

## Abstract

Cartilage grafting is an interesting option for rinoplasties refinements.

**Aim:**

to compare butyl-2-cyanocrylate, compound gelatin-resorcin-formaldeyide (GRF) and suture control to determine the efficacy of these tissue glue preparations in securing grafted cartilage.

**Study Design:**

Experimental.

**Methods:**

Fifteen male adult New Zealand rabbits were submitted to a surgical procedure to harvest 6 auricular cartilage grafts from each animal. 2 of these grafts in each animal were glued together with butyl-2-cyanocrylate, 2 with compound gelatin-resorcin-formaldehyde and 2 sewn together with nylon suture. These sandwich grafts were then glued or sewn to the periosteum of the calvaria. After 2, 6 and 12 weeks, groups of 5 animals were sacrificed and histological analysis for inflammation was performed. Cartilage graft migration, adhesion and deformities of the grafts were also evaluated.

**Results:**

there was less migration of the cartilages glued with GRF than with cyanoacrylate and suture. There was no statistical difference between the 3 materials of graft stabilization in relation to the inflammatory reaction in all evaluated periods. There wasn’t detachment neither deformity in the cartilage sandwiches sewed with suture. The majority of detachment and deformed cartilages were found among those glued with cyanoacrylate. The number of deformed cartilages was directly related to the number of detached cartilages. The data were statistically significant (p< 0.05).

**Conclusion:**

the compound gelatin-resorcin-formaldehyde revealed to be a stabilization material for cartilage grafts in rabbits better than butyl-2-cyanoacrylate. The compound gelatin-resorcin-formaldeyide was also better than the suture when comparing it's fixation to the periosteum.

## INTRODUCTION

Nasal grafts are a technical solution for rhinoplasty improvement. On the other hand, they may represent the only resource available for cases in which there is structural loss of nasal support framework. When used for refining, one must consider the use of a graft versus procedure inherent complications. The most used graft material is the autologous cartilage, with mild rates of resorption, infection and extrusion^1^. Notwithstanding, one major complication is cartilage shifting, because often times its anchorage in the nasal cavity is technically challenging^2^. Often, the inflammatory process caused by the fixating material itself facilitates both graft resorption and extrusion^3^. Thus, we have been searching for an easy to use fixating material, that causes minimal tissue reaction. Such characteristics are already present in the autologous cartilage as grafting material.

## OBJETIVE

Compare the use of butyl-2-cyanoacrylate, gelatin-resorcin and formaldehyde mixture (GRF); and suturing in the stabilization of cartilage grafts in rabbits, as far as shifting, inflammatory process, adhesion and cartilage deformities are concerned.

## MATERIALS AND METHODS

**Figure 1:**
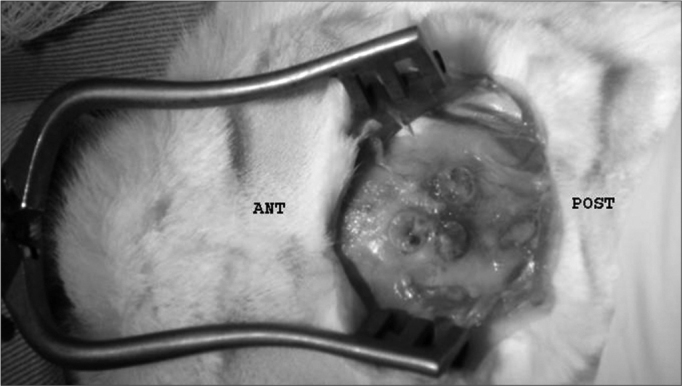
Graft location in relation to the animal.

The present study was submitted to and approved by the Medical Ethics Committee for experimental animal procedures of the Santa Casa de São Paulo. 15 adult, male, New Zealand 4kg rabbits were used, and from their ears 6 circular 5mm diameter grafts were resected. The grafts were placed two by two, bound together and fixed to the skull periosteum, each pair, with gelatin-resorcin and formaldehyde mixture (GRF) suture - Colagel ®, or butyl-2-cyanoacrylate4 - Histoacryl ®, (figure 1). After 2, 6 and 12 weeks, 5 rabbit groups were slaughtered and macroscopically studied as to the graft quantitative migration from initial implant site, and semi-quantitative histology analysis was made; and we observed inflammatory cell infiltrate, angiogenesis and fibrogenesis, besides cartilage adhesion level and their deformities.

### Procedures adopted

#### Surgery

Anesthesia: the animals were anesthetized by tiletamide chloridrate + zolazepan chloridrate (Zoletil®) in the doses of 0.4mg/kg, and fentanyl citrate+droperidol (Inoval®) at the dose of 0.3mg/kg, intramuscular injection. We then removed the right ear and skull hair, placed the animal in the operating table, under thorough antisepsis procedure. Sometimes, during the procedure, it was necessary to inject again half the anesthetic agent used during anesthesia induction. No animal required orotracheal intubation, they all spontaneously ventilated during surgery.

#### Surgical technique

**Figure 2:**
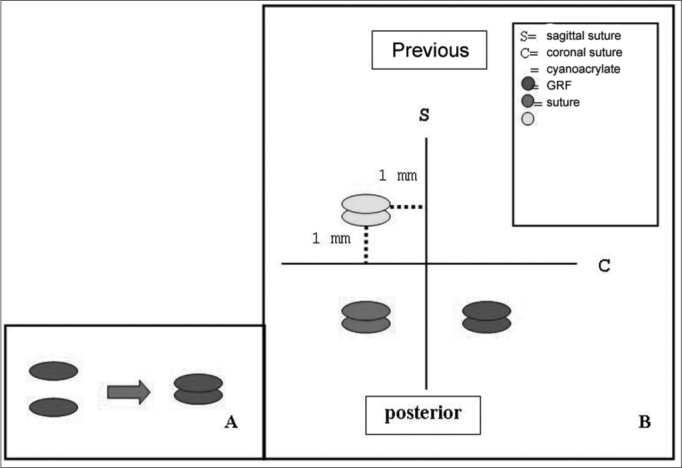
**A-** Joining of the 2 cartilage pieces; **B-** rabbit auricular cartilage grafts fixating position in the skull, in relation to cranial suture lines.

We made a 2cm incision in the right ear ventral region, detaching both the skin and the pericondrium to expose the cartilage. Following that, we resected 6 circular 5mm diameter auricular cartilage pieces and closed the incision. The cartilages were grouped two by two, and each set of two cartilages were bound by a different material (suture, GRF and cyanoacrylate). Later on a “T”-shaped incision was made on the skull, exposing the intersection of both sagittal and coronal cranial sutures. The cartilage “sandwiches” were then fixed to the cranial bone periosteum, 1 cm away from the aforementioned sutures, one in each quadrant, with the same fixating material used before to join them. Thus, in the lower left quadrant we fixed the cartilage group in which we used GRF, the lower right quadrant received the cyanoacrylate group, and the left anterior quadrant received the suture. For the sutured grafts we used 5.0 mononylon 5.0, a “U”suture and then the whole set was fixed to the periosteum by another “U” suture, the knots were placed superiorly to the cartilages (Figures 1 and 2).

#### Care

We used G procaine Penicillin (0.1ml/kg) as prophylactic agent, starting on the immediate post-op for 3 days) and dipirone for pain control until the 3^rd^ post-operative day. The animals were fed and cleaned by the Santa Casa de São Paulo Animal Lab employees.

#### Follow up

**Figure 3:**
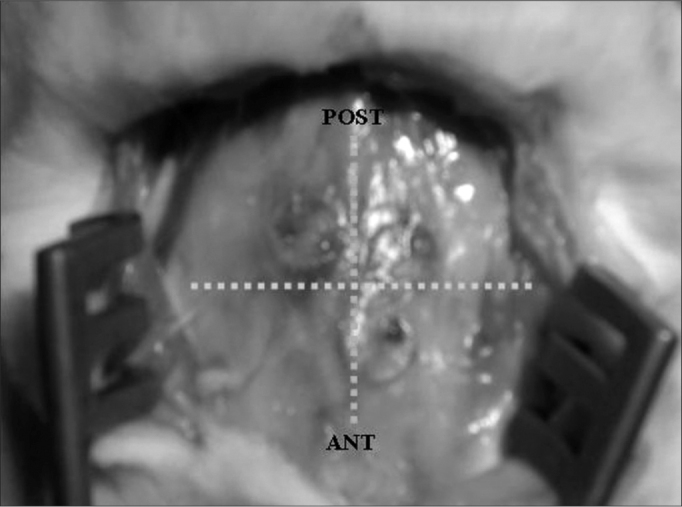
Grafts in relation to cranial suture lines (12 weeks PO).

• Follow up time for animal slaughtering was established at 2, 6 and 12 weeks.
• Quantitative macroscopic analysis of the graft migration level. The analysis of graft migration level was determined by the degree of shifting in relation to cranial suture lines. Each graft was fixed at 1mm of the cranial sutures. The shifting measuring was carried out tracing an imaginary line from the graft central point to the cranial suture, perpendicular to the latter, and measuring the distance from the border to the graft suture. From the value found, we subtracted 1mm corresponding to the initial fixing site (Figures 2 and 3).
• Material resection for histology studies

#### Time

2, 6 and 12 weeks after surgery.

#### Surgical technique

**Figure 4:**
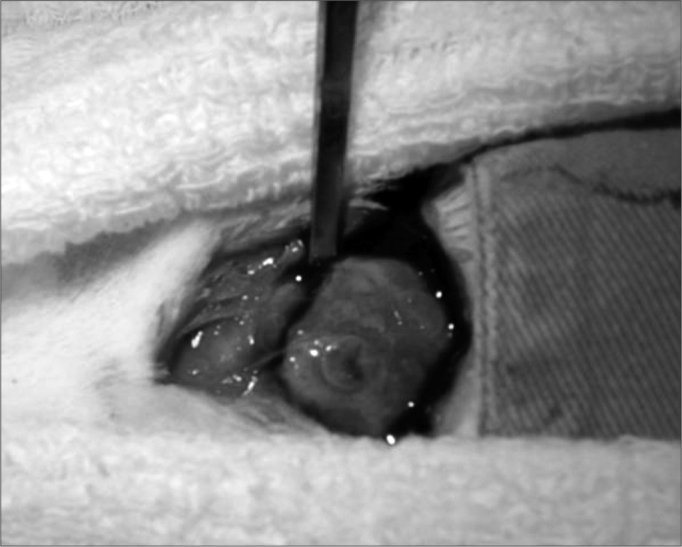
Graft block, periosteum and cortex resection with the osteotome.

**Figure 5:**
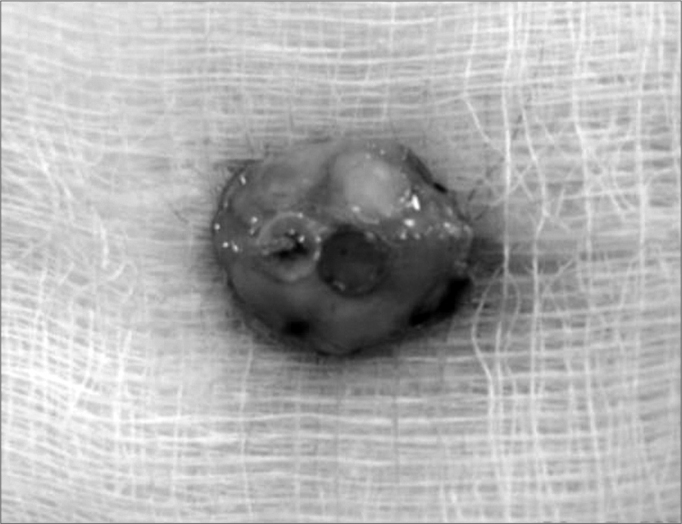
Anatomy specimen for histology analysis.

A new “T”-shaped incision was made on the animal scalp and the cortical bone was removed together with periosteum and graft, using an osteotome and a surgical hammer. The material was submerged in a formaldehyde solution for 48 hours (Figures 4 and 5).

#### Anatomy specimen preparation and histology study

Preparation: the specimens were decalcified in a 10% nitric acid solution for approximately 6 days. The solution was changed daily. They were then cut and placed in paraffin. 5 micromere cuts were made and dyed with hematoxylin-eosin.

#### Semi-quantitative histology

The following inflammatory response-related parameters were assessed – mononuclear and neutrophyl cellular infiltrates, angiogenesis and fibrogenesis. These histology findings were classified with scores from 0 to 3, according to the intensity of tissue inflammatory response (0= absent; 1= mild; 2= moderate; 3= severe). We also assessed the adhesion of cartilage pieces to themselves; between them and the cranial bone, and their shape (equal to the original shape or deformed).

#### Statistical analysis

We carried out comparative analysis using Fisher exact, qui-squared and Kruskal-Wallis tests, with a p<0.05 being significant. For the shifting analysis we considered medial horizontal shifting as positive and central vertical shifting as negative. Shifting distance average was calculated using the following formula: D=v (distance from the cartilage to the sagittal suture + cartilage to the coronal suture distance)

## RESULTS

The animals remained healthy during the studies; no surgical wound infection was seen.

### Graft shifting (migration)

**Graph 1:**
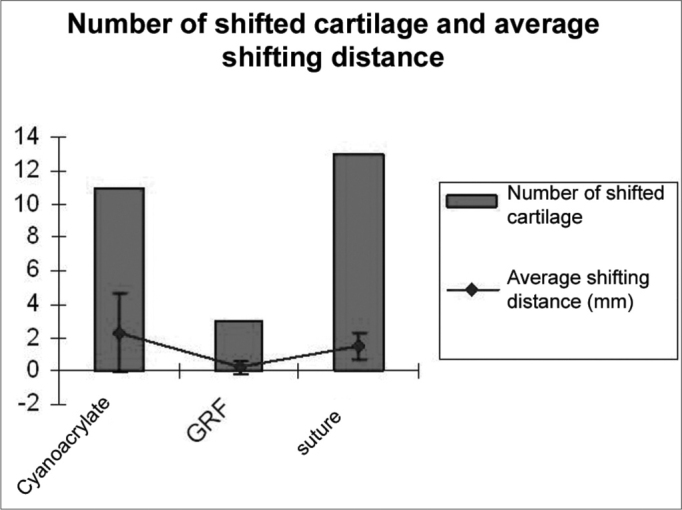
Comparison among cyanoacrylate, GRF and suture in relation to the number of shifted cartilage and the average shifting distance.

**Chart 1:**
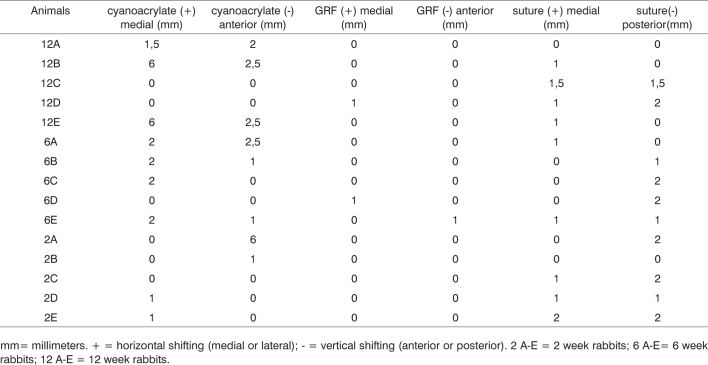
Ear graft shifting grade (in millimeters) in relation to animal cranial sutures.

Graft shifting distance assessment is depicted on Graph 1 and Chart 1. We observed that cartilage shifting pattern had a concentric fashion (the grafts tended to move towards the intersection of the sagittal and coronal cranial sutures). The number of cartilages that moved was lower in the GRF fixed cartilages when compared to cyanoacrylate and suture (p=0.0004 according to qui-squared test). There was no statistically significant difference between cyanoacrylate and suture as far as the number of shifting grafts is concerned. Comparing shifting distance averages among the three types of fixating materias, it was shorter in the GRF fixed grafts then that of the other materials (statistically significant according to Kruskal-Wallis test, p=0.0003). We noticed that although there were more suture-fixed shifted cartilage, these had a short shifting distance, up to 2mm, and the ones fixed with cyanoacrylate, although in a fewer number, did shift more (up to 6mm). However, there was no statistically significant difference between cyanoacrylate and suture in regards to shifting distance. There were no statistically significant differences between graft shifting and graft dwelling time. We could see that GRF fixed graft shifting started later on, at about the sixth week, however there was no statistically significant difference for this parameter.

mm= millimeters. + = horizontal shifting (medial or lateral); - = vertical shifting (anterior or posterior). 2 A-E = 2 week rabbits; 6 A-E= 6 week rabbits; 12 A-E = 12 week rabbits.

### Histological analysis

**Chart 2:**
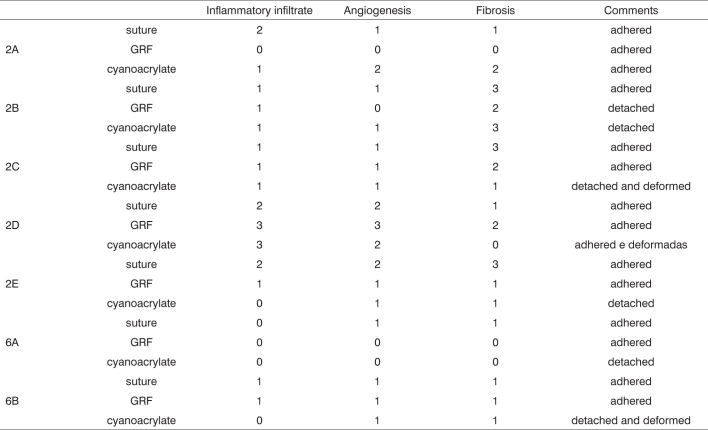

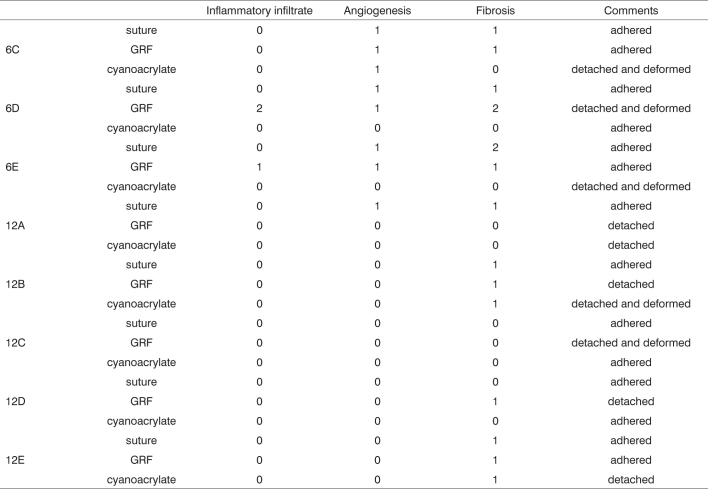
Cyanoacrylate, GRF and Suture graft fixating local inflammatory response histology grading, and deformity grade with cartilage adhesion evaluation.

**Figure 6:**
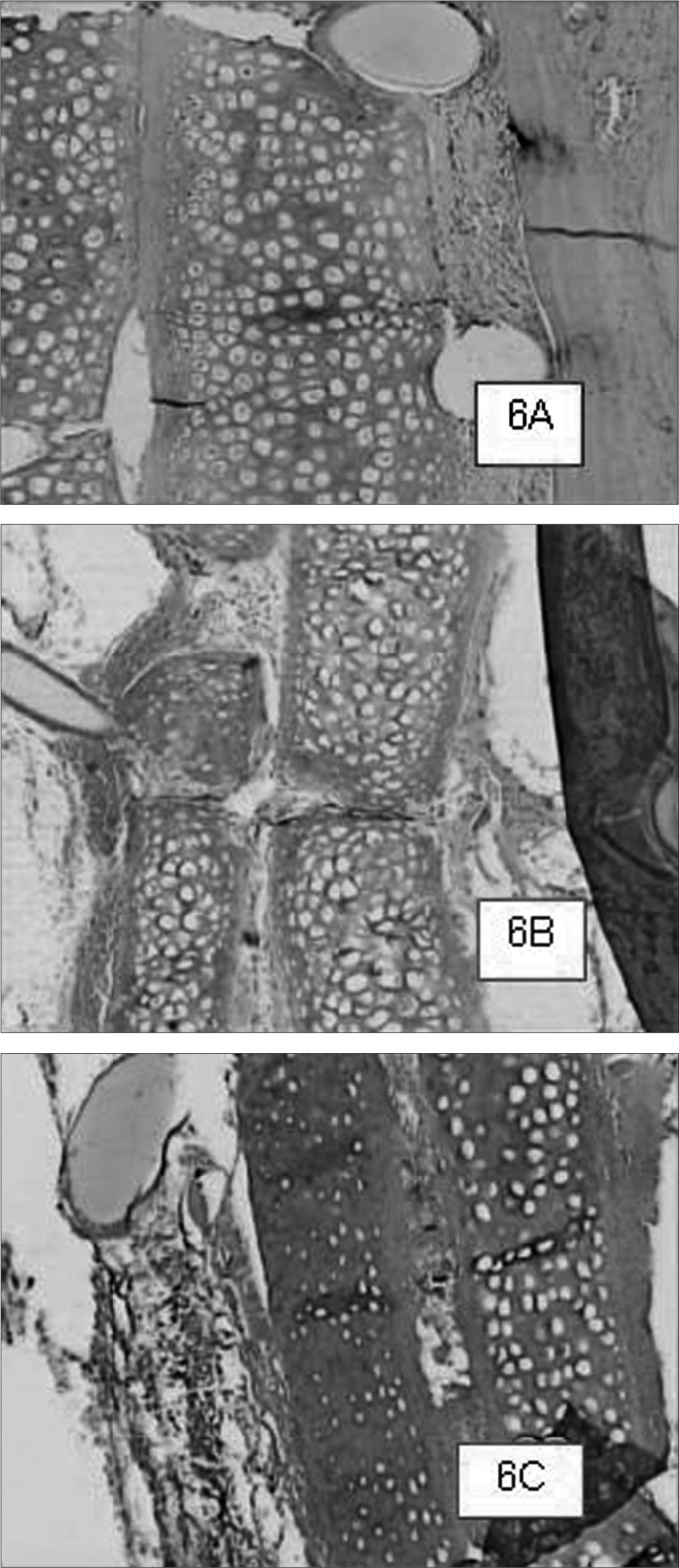
Suture-bound cartilage fragments in animals slaughtered within 2 (6A), 6 (6B), and 12 (6C) weeks after the procedure, respectively. We see a good adhesion between the cartilage and minimum inflammatory infiltrate in all cases .HE 50X A.0.

**Figure 7:**
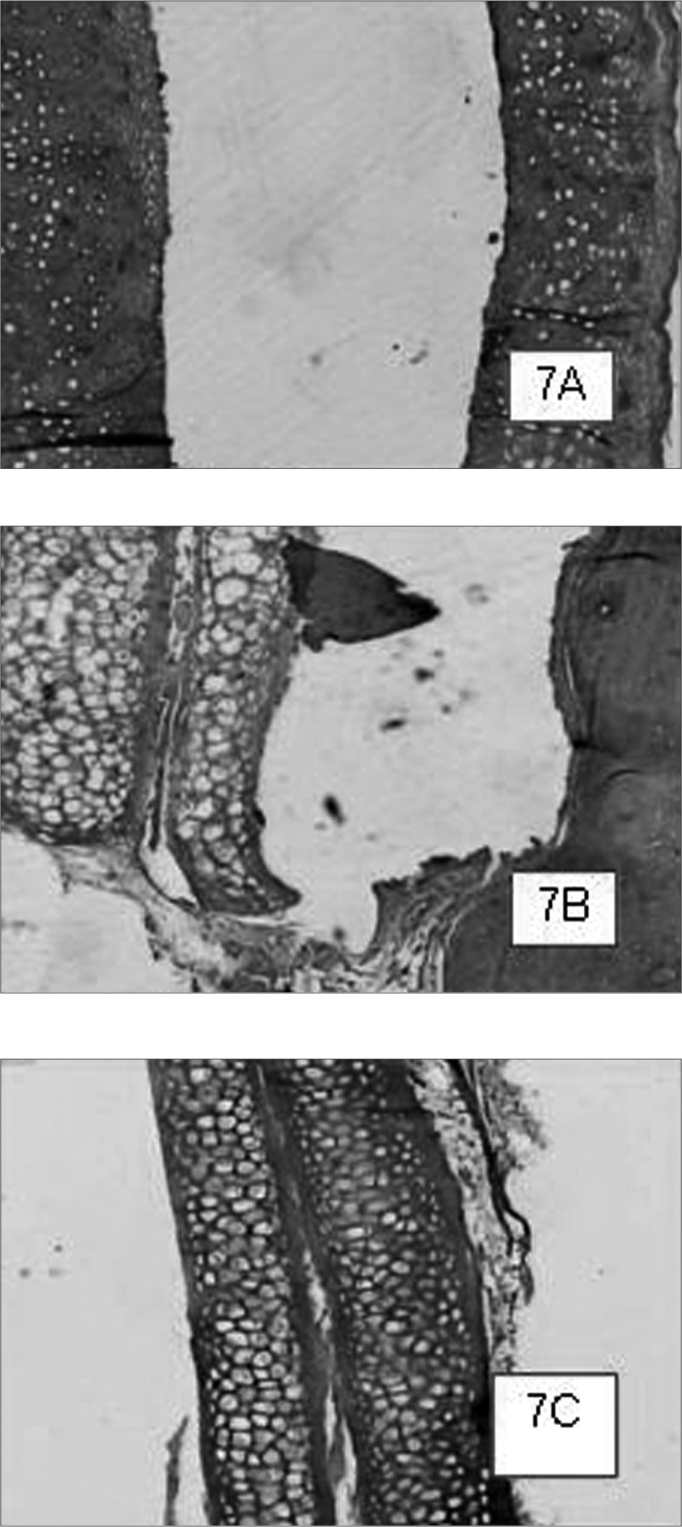
Cyanoacrylate-bound cartilage fragments in animals slaughtered within 2 (7A), 6 (7B) and 12 (7C) weeks after the procedure, respectively. In case 7A, within 2 weeks we see the cartilage fragments detachment, and in case 7B we see the shifting between cartilage sandwich and the cranial bone. HE 50X A.O.

**Figure 8:**
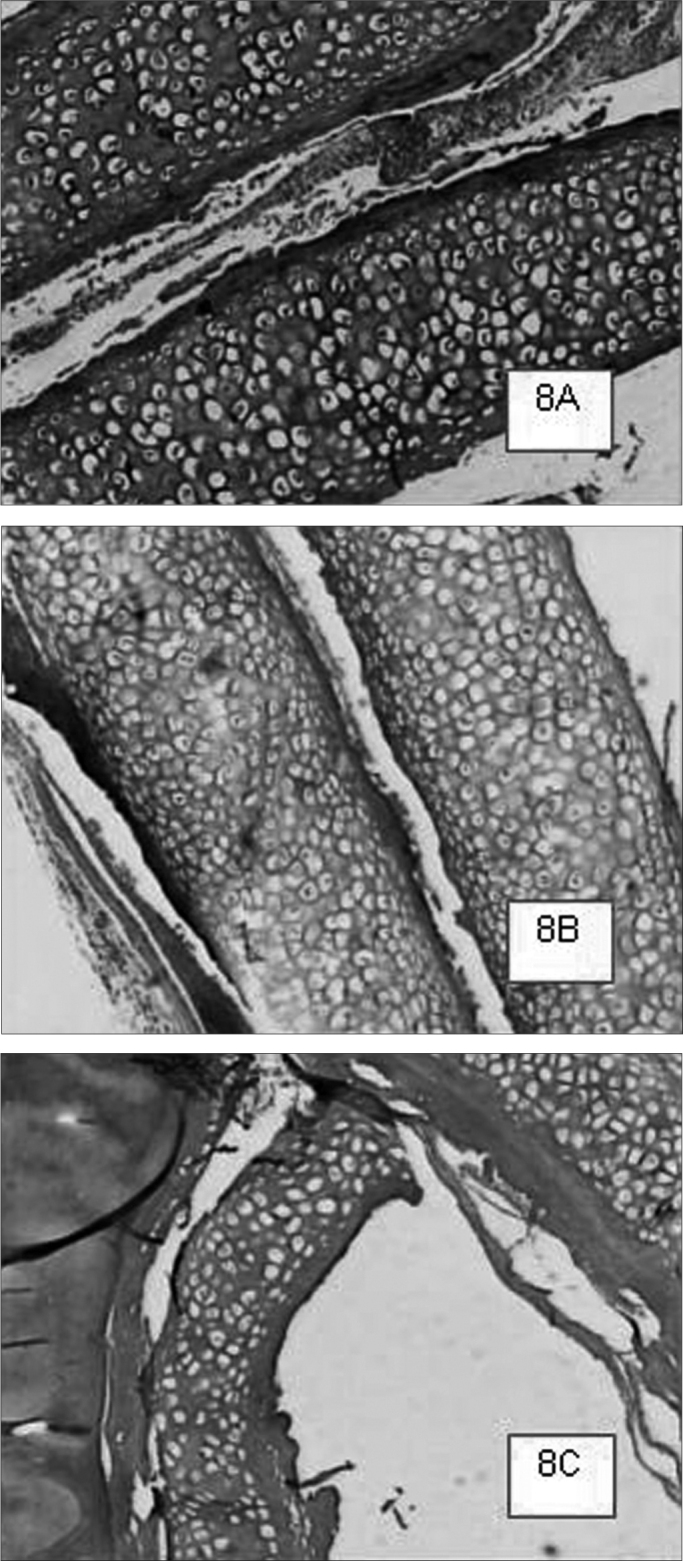
GRF-bound cartilage fragments in animals slaughtered within 2 (8A), 6 (8B) and 12 (8C) weeks after the procedure, respectively. Cases 8A and 8B show good cartilage adhesion; however, in the animal slaughtered after 12 weeks, there was cartilage detachment and deformity. We notice a minimum inflammatory infiltrate, especially in the 2 week case (8C). HE 50X A.O.

**Figure 9:**
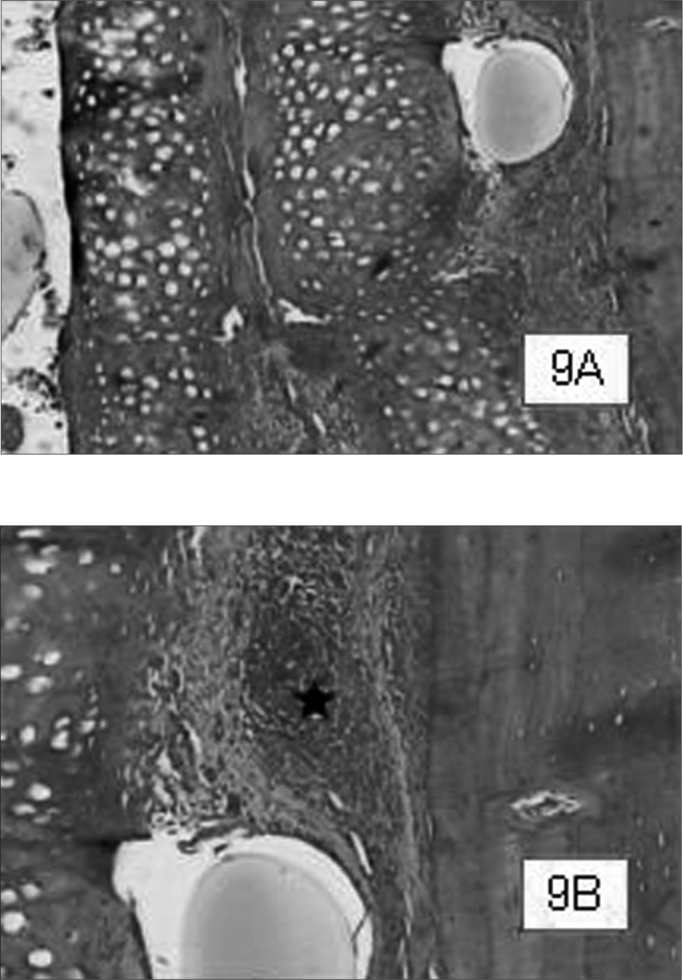
Suture-bound cartilage fragments in animals slaughtered within 2 weeks. Notice the good adhesion between cartilage fragments, and between the “sandwich” and the cranial bone. In greater magnification (9B), moderate presence of inflammatory infiltrate, besides vascular neoformation (). HE 50X e 100X A.O.

**Figure 10:**
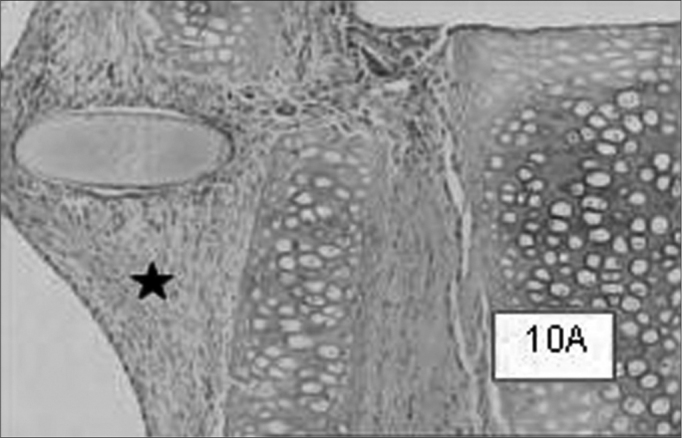

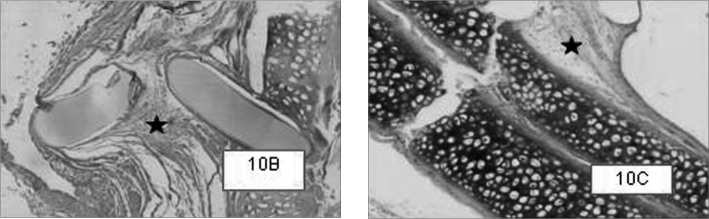
Notice the fibrosis site located around the suture wire in cases with 2, 6, and 12 weeks (10A, 10B and 10C respectively). HE 50X A.O.

**Graph 2:**
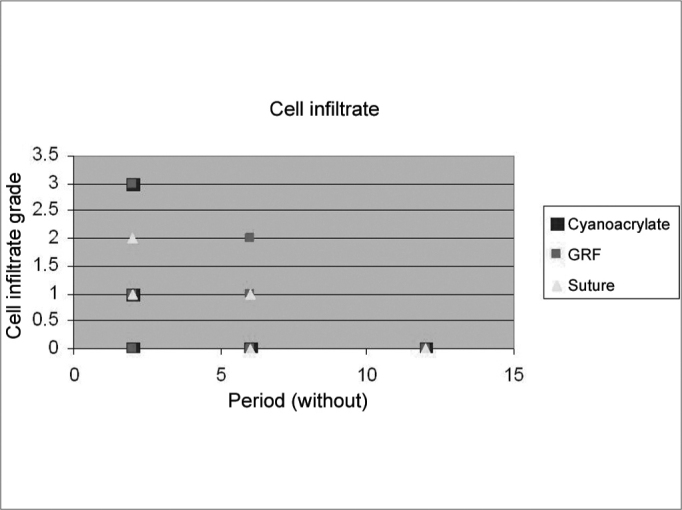
Cell inflammatory infiltrate grade in fixings with cyanoacrylate, GRF and suture in the periods of 2, 6 and 12 weeks.

**Graph 3:**
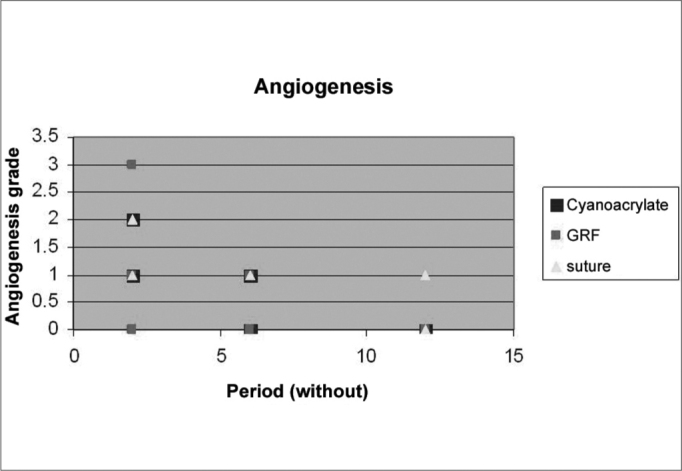
Angiogenesis grade in suture, GRF and cyanoacrylate in 2, 6 and 12 weeks.

**Graph 4:**
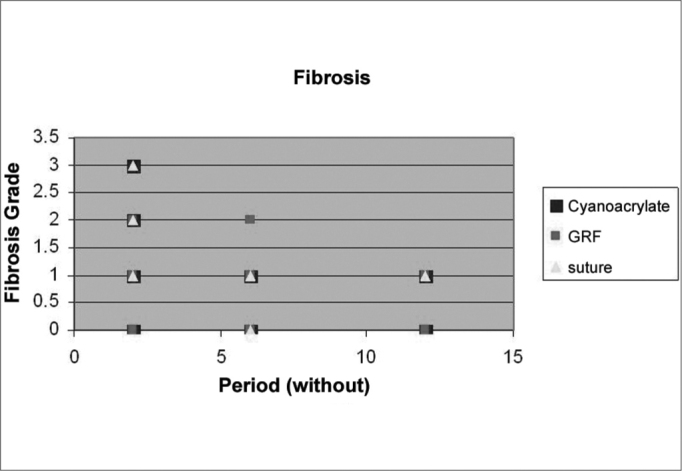
Fibrosis grade in suture, GRF and cyanoacrylate fixings in 2, 6 and 12 weeks.

Graft site histology is depicted on Chart 2 and Figure 6, Figure 7, Figure 8, Figure 9, Figure 10. The three tissue inflammatory process parameters were more intense in the animals slaughtered after 2 weeks, and the reaction intensity tapered off until there was only a mild fibrosis after 12 weeks post-op. When the different types of fixating materials were compared, there was no statistically significant difference between animal groups from 2, 6 and 12 weeks in regards of inflammatory infiltrate, angiogenesis and level of fibrosis (qui-squared test). Apparently, there was a higher grade of inflammatory process related to suturing, and less with cyanoacrylate when compared to the others, but this difference was not statistically significant (Graphs 2, 3 and 4).

### Cartilage adhesion and deformities

**Graph 5:**
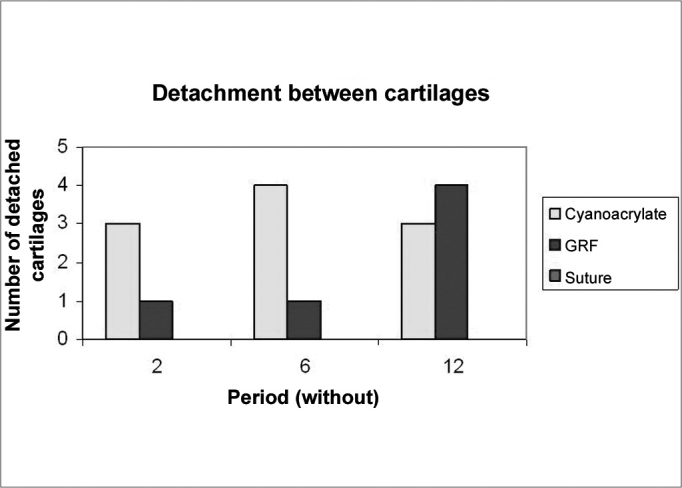
Cyanoacrylate, GRF and suture comparison of the number of detached cartilage between each other in 2, 6 and 12 weeks.

**Graph 6:**
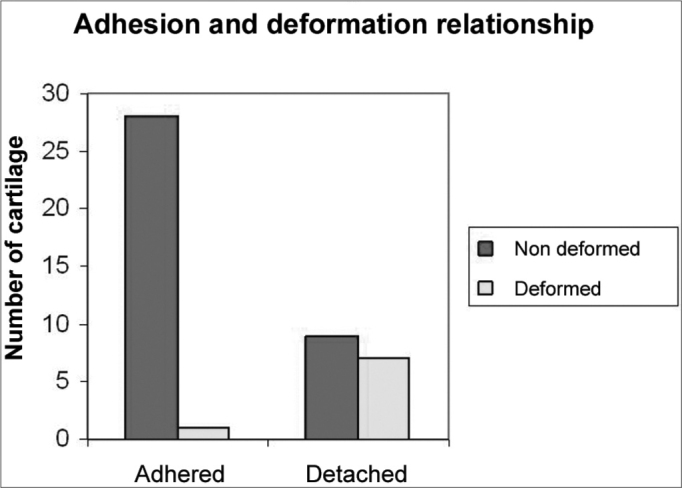
Adhesion and deformity relationship observed in the histology analysis of cartilage pieces previously fixated with cyanoacrylate, GRF and suture.

As for cartilage adhesion, depicted on Graph 5, there was no detachment between any of the cartilage pairs fixed with suturing, and the highest number of cartilage detachment happened with cyanoacrylate (statistically significant data in the 6 p=0.02 and 12 p=0.03 week animals). As to detachment time, in two weeks, the cyanoacrylate group already showed a large number of cartilage detachments (p=0.09), and in those bound by GRF, such thing only happened after 12 weeks (p=0.05). This cartilage adhesion/deformation ration observed in histology with cyanoacrylate, GRF and suturing is depicted on Graph 6. We did not observe any deformity in any cartilage fixed with cyanoacrylate, however there was no statistically significant difference among the 3 groups in the three periods. In the 16 detached cartilage groups, 7 were deformed, while in the 29 groups of adhered cartilages, only one was deformed. The Fisher Exact Test showed an adhesion/deformity statistically significant ratio (p=0.0016).

## DISCUSSION

Despite countless attempts to use synthetic material in repairing deformities, the autologous graft has proven to be the most efficient, with less resorption, less infection and less extrusion. Notwithstanding it still bears relative movement as complication^2^. In rhinoplasties, the operation field available to fix grafts with sutures is often times too narrow, and thus we search for a fixating material that is as cost-effective as suture wires. Surgical adhesive agents are usually polymers made up of addition processes or simple condensation and low molecular weight compounds, known as monomers^5^. The gelatin-based adhesive: resorcin and formaldehyde (GRF) is made up of two parts: a viscous one (gelatin) and a liquid one (polymerizing agent), and polymerizes in two minutes. Besides being of low cost, they have flexibility, low toxicity after polymerized, and biodegradability. However, its drawbacks are low transparency and the need to use free formaldehyde for polymerization^5^. Currently, GRF is broadly used in vascular surgery^6^, ^7^ and has proven successful in fixing tooth^8^. However, according to some experimental studies^5^, ^9^, ^10^ it did not prove to be efficient in ophthalmology tissue repair. Other types of broadly used surgical adhesive agents are cyanoacrylate-based polymers. In 1949, cyanoacrylate alkyl acids were made and, 10 years later, accidentally their adhesive powers were discovered. Its first commercial name was coined: “Eastman 910 Monomer”11. Cyanoacrylate long chain spin offs, such as butyl-2-cyanoacrylate (Histoacryl®) and n-butyl cyanoacrylate (Nexaband®) are degraded in cyanoacetate and formaldehyde slowly enough in order to properly reduce its citotoxic effects^12^. Cyanoacrylate polymerization causes the hardening of cyano-alkyl esters, which is responsible for both its adhesive capacity and its little elasticity. Tissue adhesiveness is brought about by an anionic mechanism triggered by water or free electrons. This polymerization is usually complete in 10 to 60 seconds^13^, ^14^. Cyanoacrylate inherent inflammatory reaction is caused by tissue oxygen-dependent reactions, instead of through the previous theory of polymerization exothermic reaction, explained by hidroperoxide lipid cell membranes polyunsaturated fatty acid transformation, which increase local arachidonic acid metabolism, triggering thromboxane and prostaglandin synthesis^15^. Cyanoacrylate proved to be an efficient adhesive for blood vessels^16^, ^17^, corneas^10^, skin^18^, cartilage-bone^4^ and other organs. Analyzing cartilage shifting in relation to the skull bone, we observed a concentric migration pattern. This might be related to the sectioned muscle action, or tissue healing pattern, as observed by Brown (1996)^4^, who used the same experimental model that we did. The results on the number of shifted cartilage and shifting distances averages show greater GRF cartilage-bone efficacy when compared to the other methods. There was no difference between butyl-2-cyanoacrylate and bone-cartilage fixating suture, in agreement with Brown (1996)^4^. Tissue inflammation analysis revealed an initially moderate inflammatory process, which tapered off until practically none in 12 weeks. There was no difference as far inflammation level is concerned among the three fixating modes, contrary to studies that showed greater GRF tissue reaction, supposing a formaldehyde-caused irritation^5^, ^12^, ^17^. Apparently, suturing caused the greater reaction (around the wire) and cyanoacrylate the least (without statistical significance). As to the adhesion of cartilage pieces, suturing indubitably proved to be the best fixating method, not allowing any cartilage pair to detach. In these grounds, GRF was also better than cyanoacrylate, with less detached rabbit cartilage from 2 to 6 weeks. One interesting aspect was the GRF pattern change in 12 weeks, with a considerable increase in the number of detached cartilage (from 1/5 in 2 to 6 weeks to 4/5 in 12 weeks), and even then not worse than cyanoacrylate. Ferrigno (2003)^17^ observed GRF adhesiveness for 30 days in rabbit venoraphe, but we still lack longer studies. The number of deformed cartilage was directly proportional to the number detached ones (no suture-fixed cartilage was deformed). Considering the aspects studied, and the fact that adhesive use is faster and safer than conventional suturing techniques, without having to use needles and of easy handling within small surgical spaces, the gelatin-resorcin-formaldehyde mixture proved to be a better rabbit cartilage graft stabilization than butyl-2-cyanoacrylate in all fixings than cartilage-bone fixating suture. In the cartilage-cartilage fixating, it proved good adhesion for up to 12 weeks. Future clinical studies will allow proving both the safety and the efficacy of such adhesive substance in rhinoplasties, malar augmentations and other facial cosmetic procedures.

## CONCLUSIONS

1. The number of shifted grafts and the average graft shifting distance were lower in GRF fixating when compared to butyl-2-cyanoacrylate and suture.
2. Tissue inflammatory reaction analysis revealed an initially moderate inflammatory process which tapered to almost zero in these animals within 12 weeks. There were no differences among the three fixating methods as far as inflammation is concerned.
3. There was no detachment of any cartilage pair fixed with suture.
4. The number of cartilages deformed was directly proportional to the number of shifted cartilage.
5. The gelatin-resorcin-formaldehyde mixture proved to be a better rabbit cartilage graft stabilization than butyl-2-cyanoacrylate and cartilage-bone suturing in all fixings.

## THANKS

Eliana Neto de Oliveira, Histology technician – Department of Morphology - Faculdade de Ciências Médicas da Santa Casa de São Paulo, for preparing hystology material. Ting Hui Ching, from the department of Biostatistics – Medical School - Santa Casa de São Paulo, for his help in the statistical analysis.
